# Sustainable extraction of phytochemicals from *Mentha arvensis* using supramolecular eutectic solvent via microwave Irradiation: Unveiling insights with CatBoost-Driven feature analysis

**DOI:** 10.1016/j.ultsonch.2025.107300

**Published:** 2025-03-04

**Authors:** Zubera Naseem, Muhammad Bilal Qadir, Abdulaziz Bentalib, Zubair Khaliq, Muhammad Zahid, Fayyaz Ahmad, Nimra Nadeem, Anum Javaid

**Affiliations:** a Department of Textile Engineering, National Textile University, Faisalabad 37610, Pakistan; b Department of Chemical Engineering, College of Engineering, King Saud University, P.O. Box 800, Riyadh 11421, Saudi Arabia; c Department of Materials, National Textile University, Faisalabad 37610, Pakistan; d Department of Chemistry, University of Agriculture Faisalabad 38040, Pakistan; e Department of Applied Sciences, National Textile University, Faisalabad 37610, Pakistan; f Shanghai Jiao Tong University Minhang Campus School of Materials Science and Engineering, China

**Keywords:** Deep eutectic solvent, *Mentha arvensis*, Sustainable, CatBoost, Feature importance, Partial dependence analysis

## Abstract

The present study revealed the higher extraction potential of sustainable choline chloride (ChCl) and ethylene glycol (EG) based deep eutectic solvent (DES) from *Mentha arvensis* via microwave irradiation. The categorical boosting (CatBoost) machine learning model was applied to optimize the extraction process against time (4–8 min), microwave power (160–320 W), and biomass quantity (1–2.0 g/10 mL) with DES. The experimentally optimized TPC 124 ± 4.0  mg GAE/g, TFC 79 ± 3.0  mg QE/g, and DPPH radical inhibition 90 ± 4.0 % evaluated in 6 min at 240 W with 1.0 g biomass. The lowest average relative errors of 0.402 % (TPC), 0.863 % (TFC), and 0.597 % (DPPH) for train and 0.679 % (TPC), 0.685 % (TFC) and 0.480 % (DPPH) for test data showed the consistency with the predicted values. The partial dependence and feature importance revealed the contributing impact of parameters for optimizing the extraction. The average contribution percentage of each predictor to the responses revealed that time contributed 32.5 % (TPC), 35.9 % (TFC), and 18.6 % (DPPH); microwave power contributed 26.7 % (TPC), 25.5 % (TFC), and 44.2 % (DPPH); while biomass contributed 40.8 % (TPC), 38.6 % (TFC), and 37.2 % (DPPH). The significant antibacterial (*S. aureus* = 25.5 ± 1.4 mm and *E. coli* = 23.5 ± 1.4 mm) with MICs (*S. aureus* = 50 ± 2.5 µg/mL and *E. coli* = 100 ± 1.5 µg/mL) and antifungal potential (*F. solani* = 22.5 ± 1.4  mm, *A. niger* = 23.5 ± 0.8 mm), with MIC (*F. solani* = 100 ± 0.4 µg/mL and *A. niger* = 50 ± 0.5 µg/mL) of optimized extracts recorded by DES. The DES would be the best alternative to traditional organic solvents based on higher extraction efficiency and sustainability.

## Highlights

• Choline chloride and ethylene glycol based DES revealed higher extraction potential of phytochemicals from *Mentha arvensis.*
• The CatBoost model was applied to optimize and analyze the phytochemicals and antioxidant potential.
• The experimentally optimized phenolics, flavonoids and antioxidant penitential showed the consistency with predicted values.
• The R2 for train, test, and complete data indicated that the CatBoost predicted the results accurately.
• The extracts of *Mentha arvensis* showed significant antibacterial and antifungal activity.

## Graphical abstract

**Figure:**
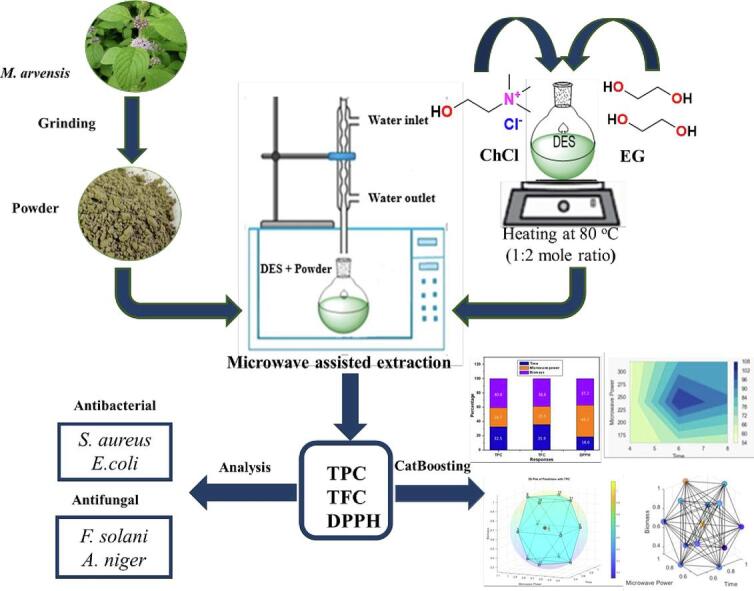

## 1 Introduction

Environmental concern has sharply increased recently, leading the chemical industry to promote eco-sustainable practices. New processes are being developed that replace conventional organic solvents like methanol, ethanol, chloroform, or hexane with biodegradable and eco-friendly solvents without compromising efficiency. Deep eutectic solvents (DESs) originated with minimal toxicity and negligible vapor pressure as substitutes for traditional harmful organic solvents [1]. Specific mole ratios of hydrogen bond donor (HBD) and acceptor (HBA) components are combined to create eutectic supramolecular solvents [2], [3]. DESs can exist as liquids at lower temperatures because their melting points are lower than their components [4]. In DESs, quaternary ammonium salts and amino acids are typically used as HBAs, while amines, sugars, alcohols, and carboxylic acids contribute as HBDs. These solvents display eutectic behavior, defined by several interactions, including hydrogen bonds, van der Waals forces, and electrostatic interactions. These interactions are vital for forming and stabilizing eutectic solvents, contributing to their distinct properties and behavior [5], [6]. Regarding their minimum level of toxicity and non-volatility, DESs are preferable to replace conventional organic solvents for medicinal applications [7].

*Mentha arvensis*, a member of the Lamiaceae family, has been used for its medicinal and therapeutic benefits since ancient times. Scientific investigation has shown that plant extracts contain bioactive chemicals that possess antioxidant, antifungal, and antibacterial activities [8]. Natural ingredients from medicinal plants with anti-aging, anti-inflammatory, anticancer, and antibacterial effects are becoming increasingly popular in the cosmetic, food, and pharmaceutical sectors. Plant-derived compounds such as phenolics/flavonoids, saponins, and terpenoids are of great interest. The main problem is the effective and eco-friendly separation and isolation of these chemicals from plants. Bioactive ingredients, including flavonoids, polyphenols, alkaloids, and plant-based pigments, have been extracted from plants using eutectic solvents [9].

Microwave-assisted extraction (MAE) offers several advantages over conventional methods, such as faster extraction times (often 1–20 min), less solvent and energy consumption, and reduced waste release into the environment [10]. MAE used a cavity magnetron to generate electromagnetic waves that strike plant cell walls and tissues, heating and evaporating moisture in the matrix. Pressure induces swelling, structural changes in the matrix, and cell rupture, leading to enhanced solute transfer and phytochemical drain from the cells [11]. Comparable yields were obtained by MAE in 4 min as opposed to 30 min for other techniques. However, scaling up could require a substantial initial investment in specialized equipment and continuing maintenance costs. Long-term cost-effectiveness might be achieved by optimizing electricity usage and reducing expenses through batch processing. MAE efficiency varies based on solvent, microwave power, extraction duration, temperature, pressure, biomass/solvent ratio, and plant matrix properties [12].

It is crucial to examine the efficacy of solvents when developing a green extraction procedure. The use of DESs, particularly choline chloride-based DESs coupled with MAE, emerges as a promising alternative, offering a green extraction method from diverse sources [13]. Their outstanding reusability reduces waste output, making them sustainable for industrial applications. However, the impact of DESs on ecosystems varies according to their composition because some of them may be able to persist in aquatic environments and affect microbial communities. Their long-term environmental destiny and interconnections need to be further studied [14]. A comprehensive life-cycle assessment is thus required to fully understand the ecological effects of DESs. In our previous studies, the DESs showed better results than aqueous methanol in the extraction process [15], [16], [17], [18].

Traditional optimization methods sometimes encounter difficulties when dealing with complicated, high-dimensional, and non-convex problems, as they may converge slowly or become locked in local optima. Furthermore, these algorithms often require manual parameter adjustment and may struggle with noisy or dynamic data. Machine learning overcomes these confines using adaptive algorithms that model detailed patterns and relationships within data, improving convergence rates and elucidation quality [19]. It solves non-linear, time-varying, multi-source, and multi-objective problems, making it useful in research fields like medicine, healthcare, and agriculture. Machine learning decreases processing costs, and data-driven techniques allow models to adapt to changing datasets, which improves robustness and generalizability [20].

Support vector machines, decision trees, random forest, extreme gradient boosting, light gradient boosting machine and categorical boosting [21], artificial neural network [22], [23], K-nearest neighbors, naïve bayes classifier [24] are the most commonly used machine learning approaches for the extraction of bioactive agents from medicinal plants. The CatBoost machine learning model is projected to be more relevant regarding problem-solving accuracy and generalization ability. CatBoost joins symmetric decision trees according to their symmetry structures to achieve the highest accuracy with less parameter testing and training. When CatBoost replaces ordered boosting with the gradient estimation approach utilized by the conventional gradient boosting methodology, gradient estimation bias is minimized, and generalization ability is enhanced with accuracy[25].

This research explores the potential of ChCl and EG-based DES as a sustainable extraction medium to extract phytochemicals from *M. arvensis* using MAE. The impact of variables, including time, microwave power, and biomass amount, on TPC, TFC, and DPPH extraction efficiency was analyzed using the CatBoost machine learning model. The absolute and relative errors were measured in the training and testing data to check the model's accuracy. The partial dependence and feature importance were investigated for each predictor's contribution against responses. The optimized extracts of *M. arvensis* were also subjected to antibacterial assessment against *S. aureus E. coli* and antifungal assessment against *F. solani* and *A. niger* and also compared with aqueous methanol.

## 2 Material and methods

### 2.1 Chemicals and reagents

This research used the analytical grade chemicals and reagents of certified companies. ChCl (99 %) and EG (99.0 %) were purchased from Dae Jung-Korea. Methanol (99.85 %) and ethanol (99.80 %) were purchased from PubChem and Riedel–deHaen, respectively. Folin-Ciocalteu reagent (99.99 %), DPPH (99.9 %), aluminum chloride (99.99 %), Na_2_CO_3_ (99 %), and KCH_3_COO (99 %) were purchased from Sigma Aldrich. Potato dextrose agar (99 %) was purchased from TmMedia, and Nutrient agar (99 %) was sourced from HiMedia.

### 2.2 Collection and preservation of *Mentha arvensis*

*M. arvensis* (mint), after being purchased from a local market, was Identified by the Department of Botany, University of Agriculture Faisalabad, Pakistan. The whole plant, including leaves and stems, was cleansed with fresh tap water to remove dirt particles. It was then dried for 30 days in the shade at 25 ± 5 °C. The material was crushed with a grinder and sieved to a 250 µm mesh size.

### 2.3 Preparation of ChCl-EG eutectic solvent

**Fig. 1:**
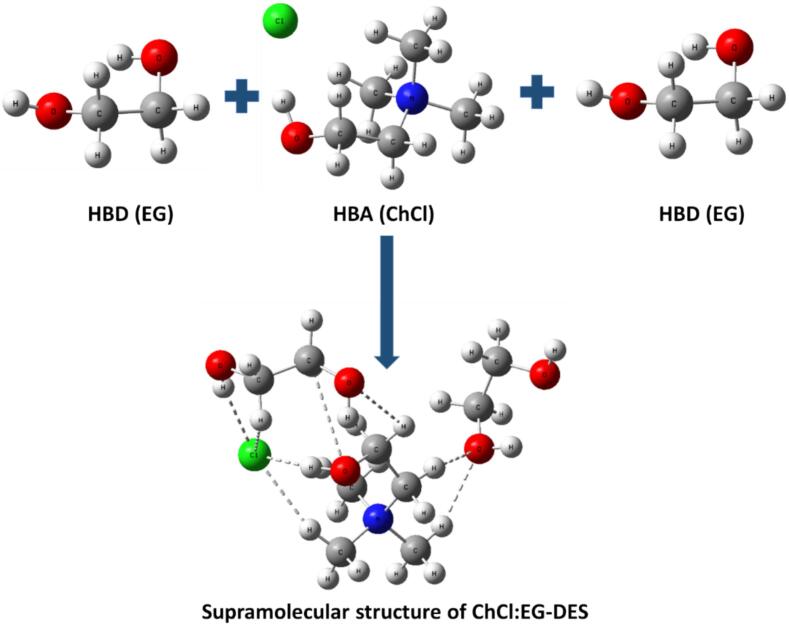
The hypothetical formation ChCl:EG-DES from ChCl (H-bond acceptor) and EG (H-bond donor) species.

1 Mole of ChCl and 2 mol of EG were chosen by reviewing the literature to prepare DES. These chemicals were desiccated in a vacuum drying oven at 60 °C overnight before use [26]. The ChCl and EG were weighed and mixed, put in a conical flask, and placed in a water bath at 80 °C until transparent and homogenous liquid appearance [15], [27]. The synthesis of DES was simple; therefore, no purification was needed after the preparation of DES. The hypothetical supramolecular structure of ChCl:EG is shown in Fig. 1

### 2.4 Extraction via microwave exposure

A modified regular microwave (Sharp R270SLM 20ltr Solo Microwave Silver) oven attached to a condenser with a maximum power output of 800 W was utilized. The dried powder of mint (1 g) and DES (10 mL) were added into a round bottom flask and kept in an oven under microwave exposure for different intervals. The sample was irradiated for 15 s and then cooled to room temperature for 60 s [28]. In MAE, the irradiation time (2–8 min), microwave power (80–320 W), and biomass quantity (0.5––2.0 g/10 mL of solvent) were selected for optimization during the initial study. The irradiation time, microwave power, and biomass quantity were chosen to optimize the yield, as these factors significantly affect the yield of phenolics during microwave-assisted extraction. Based on the optimization, further evaluation of TPC, TFC, and DPPH was carried out using the customized design of the experiment, as shown in Table 1.

**Table 1:** The experimental design for optimizing the extraction parameters (Time, Microwave Power, and Biomass) to evaluate the phytochemicals (TPC, TFC) and antioxidant (DDPH assay) of *M. arvensis* using ChCl:EG-DES.

**Index**	**Time (min)**	**Microwave Power (W)**	**Biomass (g)**	**TPC** **(mg GAE/g)**	**TFC** **(mg QE/g)**	**DDPH** **(% inhibition)**
1	6.00	160.00	0.50	61 ± 3.0	43 ± 1.5	51 ± 3.0
2	6.00	240.00	1.00	124 ± 4.0	73 ± 3.0	86 ± 4.0
3	4.00	320.00	1.00	55 ± 2.5	39 ± 1.0	68 ± 3.5
4	8.00	240.00	0.50	56 ± 2.5	23 ± 1.0	54 ± 3.0
5	4.00	160.00	1.00	56 ± 2.5	37 ± 1.0	47 ± 3.0
6	6.00	240.00	1.00	120 ± 4.0	79 ± 3.0	87 ± 4.0
7	6.00	320.00	0.50	45 ± 2.0	39 ± 1.0	57 ± 3.0
8	8.00	160.00	1.00	66 ± 3.0	28 ± 1.0	60 ± 3.5
9	6.00	320.00	1.50	91 ± 3.5	67 ± 2.5	87 ± 4.0
10	4.00	240.00	0.50	48 ± 2.0	41 ± 1.5	61 ± 3.5
11	6.00	160.00	1.50	67 ± 3.0	46 ± 1.5	56 ± 3.0
12	8.00	240.00	1.50	98 ± 3.5	51 ± 2.0	80 ± 4.0
13	6.00	240.00	1.00	121 ± 4.0	76 ± 3.0	86 ± 4.0
14	8.00	320.00	1.00	98 ± 3.5	45 ± 1.5	68 ± 3.5
15	6.00	240.00	1.00	120 ± 4.0	77 ± 3.0	90 ± 4.0
16	4.00	240.00	1.50	61 ± 3.0	49 ± 1.5	70 ± 3.5
17	6.00	240.00	1.00	118 ± 4.0	74 ± 3.0	89 ± 4.0

### 2.5 Centrifugation

Vacuum filtration assembly was used to separate the recovered components from the residual plant biomass. The filtrate was diluted ten folds with anti-solvent (water) and centrifuged (SBC0060-230 V Select Spin^TM^ Spectra-6C) at 6500 rpm for 60 min in falcon tubes to separate the DES from the phytochemicals. The precipitated bioactive compounds dried in a desiccator until they achieved a constant weight. For further examination, the dried solid components of each extract were stored at –4 °C [16].

### 2.6 Extraction yield

The yield of extracts was calculated using the following equation at optimum levels of all studied factors

$$Yield\;\left(mg/g\right)=\frac{\mathrm{weight}\;\mathrm{of}\;\mathrm{dry}\;\mathrm{extracted}\;\mathrm{material}}{\mathrm{weight}\;\mathrm{of}\;\mathrm{plant}\;\mathrm{material}}$$

### 2.7 Quantitative analysis of phytochemicals

The TPC was assessed using the modified Folin-Ciocalteau’s (FC) reagent method [29]. The TPC was measured as mg gallic acid per gram of plant dry matter (mg GAE/g). The aluminum chloride colorimetric technique was considerably modified to evaluate the TFC [30]. The TFC was measured as mg of quercetin per g of plant dry matter (mg QE/g). The DPPH radical was used to measure the extracts' proficiency to scavenge free radicals as percent inhibition, following the established biological protocol with slight modifications [31]. Supplementary data describe the testing procedures used to assess phytochemicals and their antioxidant properties.

### 2.8 Experimental data

The 3D geometrical representation of predictors (time, microwave power, and biomass) in the x, y, and z planes is presented in Fig. S1. The x, y, and z planes show the three-dimensional geometrical representation of the predictors: time, microwave power, and biomass. Each axis in this model represents a predictor: time is defined by the x-axis, the y-axis indicates microwave power, and the z-axis shows biomass. This depiction extensively examines the interactions between various factors during the extraction process. By looking at the geometrical representation, it is simpler to spot patterns, ideal circumstances, and the connections between the predictors, making it easier to comprehend how all predictors work together to affect the results.

### 2.9 Feature selection and modeling

The accuracy and efficacy of the final model will be influenced by the frequently high level of redundant information in the acquired data. Five methods, Decision Tree (DT), Random Forest (RF), Gradient Boosting (GB), Extra Trees (EX), and Categorical Boosting (CatBoost), were used initially [32], [33], [34]. The CatBoost model was selected as a foundational algorithm among all others as it handles categorical features of data efficiently. CatBoost is a robust machine-learning framework that can be applied to classification and regression tasks. Feature importance of the CatBoost model over Decision Tree, Random Forest, Gradient Boosting, and Extra Trees for all responses is shown in supplementary data as Fig. S2-Fig. S4.

### 2.10 Antimicrobial testing

This study examined the antimicrobial effects of extracts against bacterial strains, namely Gram-positive *S. aureus* and Gram-negative *E. coli* [35], as well as against fungal strains *F. solani* and *A. niger* [36] by well-diffusion assay. The Broth micro-dilution technique was used to calculate the optimized extracts' MIC (minimum inhibitory concentration). The detailed biological protocols regarding antimicrobial activity have been provided in the supplementary document.

## 3 Results and discussion

### 3.1 Optimization of independent variables based on extraction yield

***Time*:**

A preliminary investigation was conducted to determine the ideal irradiation time range for the highest phytochemical yield. To optimize the extraction time from 2-8 min, the biomass (1 g/10 mL) and microwave power (80 W) were kept constant. After 6 min of microwave irradiation, the highest yield of 276 mg/g with ChCl: EG and 179 mg/g with MeOH: H_2_O was obtained. The pressure generated by microwaves on the inner side of biomass cells ultimately improves the extraction rate in a shorter duration [37]. Multiple research studies suggest that prolonging the extraction duration leads to a higher yield of the extracted material. This highlights a common trend in extraction processes where increasing the extraction time is correlated with the greater quantity of the desired compounds or substances [38], [39], [40].However, it is important to note that excessive exposure to heat over extended periods can result in the oxidation of phenolic compounds.

***Microwave power*:** The breakdown of phenolic compounds might result from high temperatures; hence, significant consideration should be given to the microwave power selection [41]. Microwave exposure along with low to moderate power allows achieving higher purity and selectivity of phenolic extraction. This process also helps in efficiently isolating phenolics by minimizing the degradation of bioactive components which optimizes the extraction process. The use of microwave energy is controlled so that the target compounds are isolated selectively which improves both yield and quality. [42]. The bioactive components using DES and aqueous methanol were extracted under microwave power from 80–320 W with optimized time (6 min). A consistent biomass quantity of 1 g/ 10 mL was used in preceding assessments to optimize the microwave irradiation time and power. A significant yield of 312 mg/g was obtained with ChCl: EG solvent at 240 W; no appreciable yield improvement was seen up to 320 W. The yield increased gradually (279 mg/g) up to 240 W and remained constant even at 320 W with aqueous methanol. Higher microwave powers often cause a quick rise in sample temperature, making the extraction process more quick and effective [43]. Moreover, the eutectic solvents become less viscous at higher temperatures, facilitating the extraction process.

**Fig. 2:**
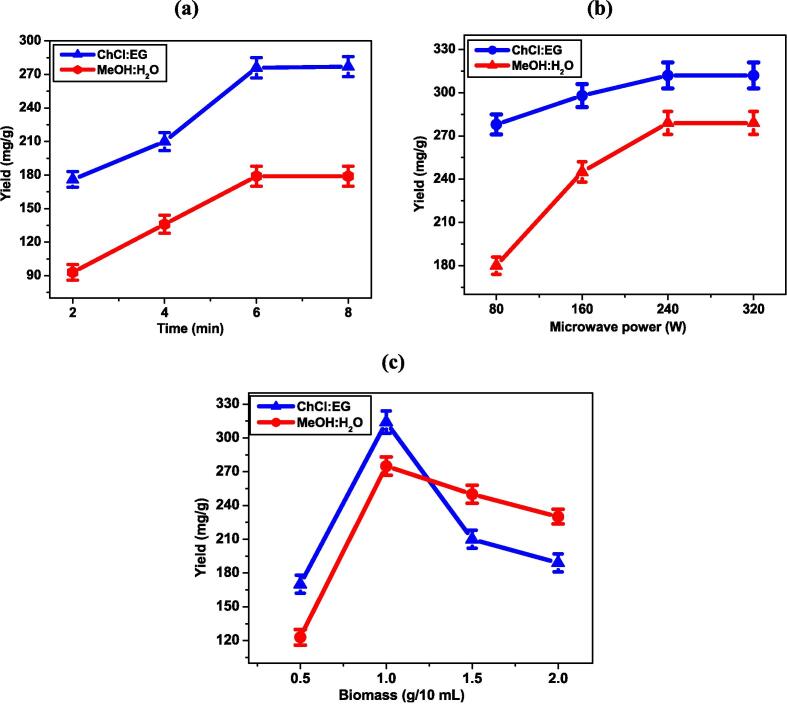
Effect of independent variables: (a) time, (b) microwave power, (c) biomass quantity on the extraction yield of phytochemicals from *M. arvensis* using ChCl:EG-DES and 80% aqueous methanol.

***Biomass amount*:** The optimized biomass quantity per unit volume of solvent is favorable for extracting the maximum phytochemicals from plants. A relatively small amount of biomass in the solvent might result in just a portion of the important bioactive chemicals being extracted. In contrast, an elevated level could produce considerable redundant waste material in the solvent. Increased biomass in the solvent accelerates the diffusion process of bioactive compounds; however, beyond a certain point, solute molecule saturation in the solvent causes the yield of phytochemicals to start declining, so the optimized level is mandatory for effective extraction [44]. The biomass amount ranging from 0.5-2.0 g per 10 mL was selected for both solvents to extract the phytochemicals. The yield of phytochemicals with ChCl: EG was increased from 0.5 g to 1.0 g (170–314 mg) and then decreased from 1.0 to 2.0 g. Similarly, the yield of phytochemicals with MeOH:H_2_O was increased from 0.5 g to 1.0 g (123–275 mg) and then declined from 1.0 to 2.0 g. The results of the optimization of time, microwave power, and biomass optimization are shown in Fig. 2(a, b, and c).

The solvent's ability to dissolve is a significant factor in determining how well an extraction process works. Since phenolic compounds are usually polar, the type of solvent used in their extraction is quite important. Polar solvents often extract more phenolics and flavonoids than less polar or nonpolar solvents [16]. A strong hydrogen bonding network (Fig. 1) and polarity of eutectic solvents increase the extraction efficiency of phenolics and flavonoids by increasing dissolution levels [45].

### 3.2 Machine learning prediction models for predicting the phytochemicals and their antioxidant potential

Polynomial regressions are among the simplest models for making predictions and are highly interpretable due to their linear nature in the coefficient space, resulting in low computational costs.Generalized inverses can be used to manage the ill-conditioned matrices of polynomial features. However, polynomial regressions need to capture the complex nonlinearities in the data. Machine learning algorithms best address this hidden complexity when the relationship between features and the response is highly non-linear and complex [46]. Both types of models are significantly dependent on the qualitative aspects of processing data. Prediction models are reliable when the data is accessible from outliers and feature correlations; otherwise, their reliability is questionable. It is crucial to ensure data cleanliness using outlier detection techniques before building prediction models based on polynomial regressions or artificial intelligence. For instance, outlier-robust extreme learning machines (ELM) and robust fuzzy regression functions (FRFN) enhance robustness against outliers [47], [48]. One can use linear and rank correlation coefficients to detect correlations between features. If the correlation is high, dependent features may be dropped, and principal component analysis (PCA) may be employed [49]. Furthermore, robust regression methods like the robust regularized extreme learning machine using incrementally reweighted least squares (RELM-IRLS) and the Wilcoxon-norm-based robust extreme learning machine (WRELM) offer improved accuracy and stability in the presence of outliers and multicollinearity [50], [51].

**Fig. 3:**
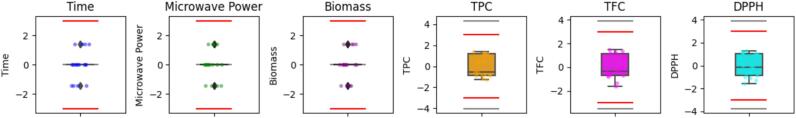
Box diagram to detect the outliers of extraction factors (time, microwave power, and biomass) and responses (TPC, TFC, and DDPH) of *M. arvensis* with ChCl:EG-DES.

An observation might be considered an outlier if it is significantly different from the other data points in a given group and its exclusion results in it being classified as a member of a distinct group. The observed data, which is unusual in nature, suggests that this particular observation is outside the expected range or distribution of the primary group. This may imply that there is a process or a variable that is different from the primary group [52], [53], [54]. The existence of outliers in predictors and responses can substantially affect the effectiveness of machine-learning systems.[55]. The z-score is a valuable tool for identifying outliers in data with a normal distribution, and the interquartile range can be used as a backup measure. Before developing a machine learning method for predicting the behavior of responses, it is crucial to analyze the relationship between the predictors after eliminating outliers from the data. The measured values of time, microwave power, biomass, TPC, TFC, and DPPH are free from outliers, as shown in Fig. 3*.*

**Fig. 4:**
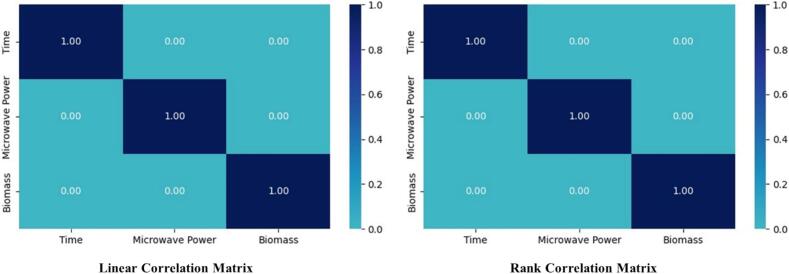
The correlation matrix shows the Spearman rank and Pearson linear correlation among extraction-dependent factors.

The correlation between the features directly impacts prediction accuracy [56]. Correlations between variables can be measured using various methods. Pearson’s correlation coefficient indicates the quantitative strength of a linear relationship between two variables, while Spearman’s rank relationship coefficient measures the relationship quality between two factors. This measure of monotone affiliation is used when information appropriation renders Pearson’s relationship coefficient unfavorable or misleading [57]. The analysis presented in Fig. 4 reveals that no significant correlations were detected among the features. The zero correlation coefficients indicate that feature dependencies will not affect prediction models.

**Fig. 5:**
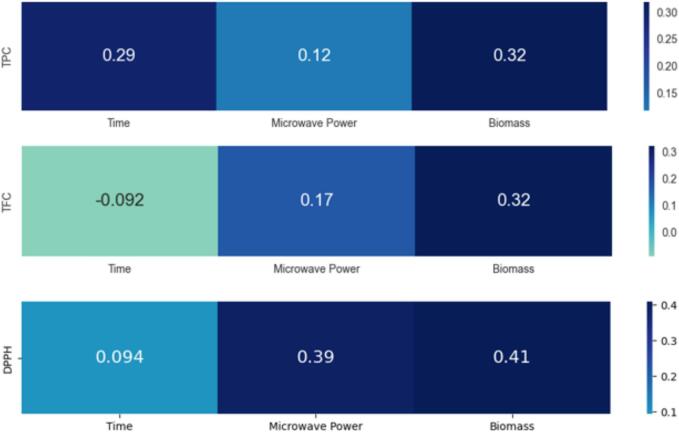
Linear correlation coefficients between the extraction predictors (Time, Microwave Power, and Biomass) and responses (TPC, TFC, and DPPH).

The linear correlation coefficients relationship between features (time, microwave power, and biomass) and (TPC, TFC, and DPPH responses) are shown in Fig. 5. TPC is reasonably correlated with time, while TFC and DPPH have near-zero correlations. The microwave ower correlation between TPC and TFC is weak, while its correlation with DPPH is comparatively better. The correlation of the biomass is high but less than 0.5 with all responses. The correlation analysis motivates us to believe there is a relationship between features and responses, and it is worth designing a prediction model between features and responses. It emphasizes how crucial it is to create a predictive model to explore these links in more detail and use them to improve our analysis's forecast accuracy. This proactive strategy is in line with the data-driven technique. It highlights the need to use these correlations to build a robust prediction model that captures and uses the interaction between the responses and predictors.

### 3.3 CatBoost machine learning model

This work uses the CatBoost model as the foundational algorithm for assessing phytochemicals and their potential as antioxidants. This model uses the t-SNE algorithm’s clustering advantage for non-linear variables to enhance model performance while retaining the CatBoost model’s excellent ability to extract feature variables and its capacity to generate high classification results without requiring a lot of data training [58]. The main characteristic of CatBoost, based on the gradient boosting decision tree algorithm framework, is its direct support for category characteristics, including string-type data. The experimental data of TPC, TFC, and DPPH (Table 1) were divided into test data and train data to train and test the model. 13 values were selected to train the model, and four values were used for testing. Machine learning methods are also used to predict ideal conditions for non-linear systems, producing the best possible results [59], [60], [61]. To build the CatBoost prediction model, we use the open-source Python library PyCaret. Table 2 describes the PyCaret setup parameter initialization for the CatBoost algorithm for all the TPC, TFC, and DPPH responses.

**Table 2:** The PyCaret setup parameters to build the CatBoost algorithm.

**Description**	**Value**	**Description**	**Value**
Session ID	123	Imputation Type	Simple
Target	TPC	Numeric Imputation	Mean
Target Type	Regression	Categorical Imputation	Mode
Original Data Shape	(17, 4)	Fold Generator	KFold
Transformed Data Shape	(17, 4)	Fold Number	5
Transformed Train Set Shape	(13, 4)	CPU Jobs	−1
Transformed Test Set Shape	(4, 4)	Use GPU	False
Numeric Features	3	Log Experiment	False
Preprocess	True	Experiment Name	reg-default-name
		USE	8671

The setup parameters show that the K-fold (5-fold) method is used to check the trained model's performance and protect the trained model from overfitting. The K-fold Table 3 shows the minimum, maximum, mean, and standard deviations of MSE and R-square values for the TPC, TFC, and DPPH responses. The minimum and maximum R-squared values are reasonable because we have only 13 observations as training data for the model.

**Table 3:** K-fold method to check the performance of the CatBoost algorithm for TPC, TFC, and DPPH responses.

	**TPC**		**TFC**		**DPPH**	
**Fold**	**MSE**	**R^2^**	**MSE**	**R^2^**	**MSE**	**R^2^**
1	226.6316	0.6824	218.5324	0.5229	125.0057	0.4658
2	232.0564	0.2662	397.6548	−1.6748	158.0910	−0.1328
3	510.8046	0.5607	60.1034	0.7047	58.5159	0.5840
4	498.0795	0.5426	223.4943	0.3470	16.8224	0.7923
5	43.6265	0.6395	6.7028	0.9738	35.7648	0.7044
**Min**	43.6265	0.5383	6.7028	0.3470	16.8224	0.4658
**Max**	510.8046	0.6824	397.6548	0.9738	158.0910	0.7923
**Mean**	302.2397	0.5383	181.2975	0.1747	78.8400	0.4827
**Std**	4.5638	0.1453	137.9484	0.9477	53.9012	0.3269

After the K-fold evaluation that we performed over the training data, we trained the model over the 13-values and kept the 4-values for the testing of the model. The measured and predicted values with absolute and relative errors of train and test data of all three responses, TPC, TFC, and DPPH, are shown in Table 4. A slight variation is present between the experimentally measured values and the predictions produced by the model. The absolute errors, whether observed in the training or testing data set, are minimal. This indicates that the model's predictions closely align with the actual measured values, suggesting high accuracy and reliability in the model's performance across the analysis's training and testing phases. The study reported minor average relative errors for TPC, TFC, and DPPH of 0.402 %, 0.863 %, and 0.597 % in the training dataset, while in the testing dataset, corresponding values of 0.679 %, 0.685 %, and 0.480 %, respectively were observed. These mimumal errors underscore the model's accuracy and reliability in predicting these parameters, demonstrating the potential for effective performance in practical applications.

**Table 4:** Comparison of measured and predicted values with train and test data errors for TPC, TFC, and DPPH of *M. arvensis* with ChCl:EG DES by CatBoost.

**Train data**														
**True index**	**Measured**				**Predicted**				**Absolute error**			**Relative error (%)**		
	**TPC**		**TFC**	**DPPH**		**TPC**	**TFC**	**DPPH**	**TPC**	**TFC**	**DPPH**	**TPC**	**TFC**	**DPPH**
1	61		43	51	61.072		43.042	51.076	0.072	0.042	0.076	0.118	0.098	0.149
2	124		73	86	120.60		75.80	87.60	3.400	2.799	1.599	2.742	3.835	1.860
3	55		39	68	55.114		39.08	68.031	0.114	0.080	0.031	0.208	0.205	0.045
4	56		23	54	56.06		23.12	54.069	0.063	0.126	0.069	0.113	0.549	0.129
6	120		79	87	120.60		75.80	87.60	0.599	3.200	0.599	0.499	4.050	0.689
7	45		39	57	45.145		39.051	57.048	0.153	0.051	0.048	0.342	0.132	0.084
8	66		28	60	66.096		28.102	60.017	0.090	0.102	0.017	0.137	0.365	0.029
11	67		46	56	67.076		46.028	56.051	0.076	0.028	0.050	0.113	0.065	0.091
12	98		51	80	97.992		51.003	79.972	0.002	0.003	0.027	0.003	0.006	0.033
13	121		76	86	120.60		75.80	87.60	0.400	0.200	1.599	0.330	0.263	1.860
14	98		45	68	97.994		45.031	68.057	0.005	0.031	0.057	0.005	0.069	0.084
15	120		77	90	120.60		75.80	87.60	0.599	1.200	2.400	0.499	1.558	2.666
16	61		49	70	61.068		49.014	70.031	0.068	0.014	0.031	0.111	0.030	0.045
**Average relative error**												**0.402**	**0.863**	**0.597**
**Test data.**														
5	56	37		47	56.095		37.055	47.096	0.095	0.055	0.095	0.171	0.149	0.204
9	91	67		87	91.057		66.941	86.922	0.057	0.051	0.077	0.062	0.077	0.089
10	48	41		61	48.135		41.033	61.032	0.134	0.033	0.032	0.281	0.082	0.052
17	118	74		89	120.6		75.80	87.60	2.599	1.799	1.400	2.203	2.432	1.573
**Average relative error**												**0.679**	**0.685**	**0.480**

#### 3.3.1 Regression and error plots

**Fig. 6:**
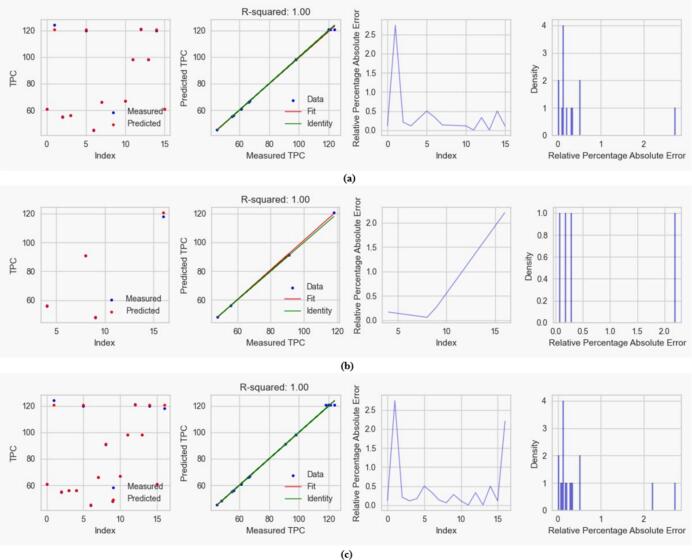
Visual representation of CatBoost on (a) train data, (b) test data, and (c) complete data prediction performance of TPC of *M. arvensis* with ChCl:EG-DES showing regression and error plots including R-squared values, predicted vs. measured TPC, relative percentage absolute error and density distributions.

**Fig. 7:**
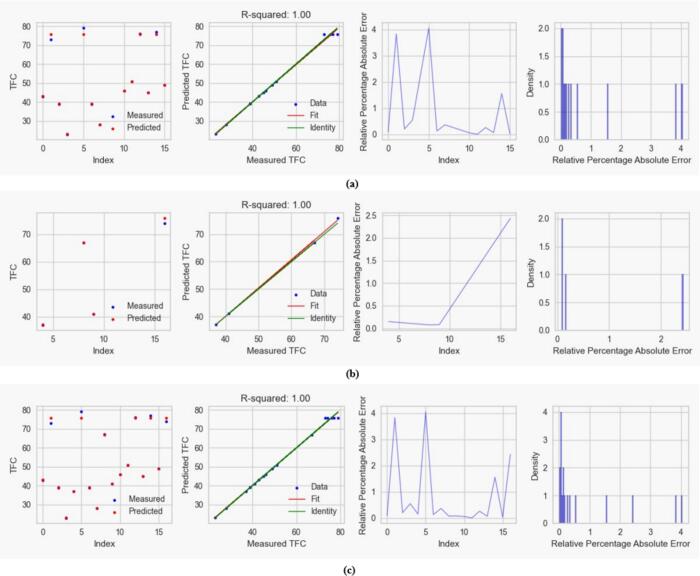
Visual representation of CatBoost on (a) train data, (b) test data, and (c) complete data prediction performance of TFC of *M. arvensis* with ChCl:EG-DES showing regression and error plots including R-squared values, predicted vs. measured TFC, relative percentage absolute error and density distributions.

**Fig. 8:**
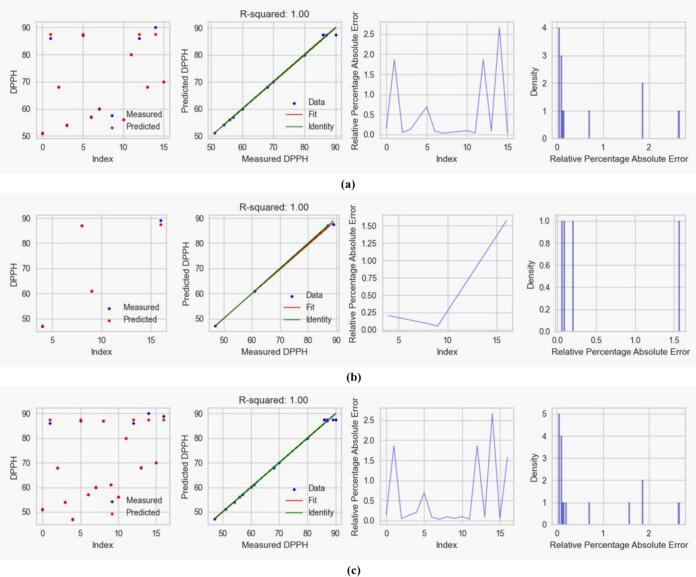
Visual representation of CatBoost on (a) train data, (b) test data, and (c) complete data prediction performance of DDPH of *M. arvensis* with ChCl:EG DES showing regression and error plots including R-squared values, predicted vs. measured DPPH, and relative percentage absolute error and energy distributions.

Accordingly, all data points fall precisely on the regression line, indicating that the regression model accurately predicts the outcome variable. Error plots show a precise relationship between the actual and predicted responses, indicating that the model explains the data's variability. Fig. 6, Fig. 7 and Fig. 8 shows the scatter plots, R-squared values, frequency bar chart of relative percentage absolute errors and density distributions for training, testing, and complete data sets. The relative percentage errors for the TPC, TFC, and DPPH responses are less than 0.5, 1.0, and 0.6, respectively. In all the cases, the R-squared values for the training, testing, and complete data set are one, showing that the CatBoost models explain the data well.

#### 3.3.2 Partial dependence study

**Fig. 9:**
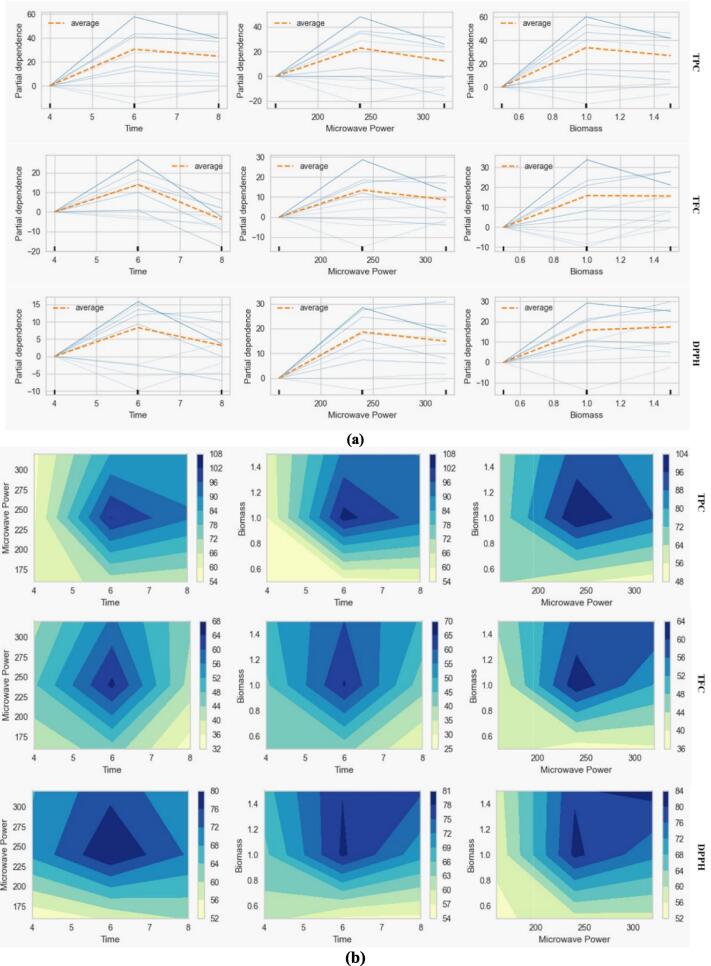
The 2D illustration (a) and partial dependence plots (b) cumulative impact for the feature effects on the predictions for TPC, TFC, and DPPH of *M. arvensis* with ChCl:EG- DES.

The effects of independent variables on the average of predicted responses are displayed in partial dependency plots, as shown in Fig. 9 (a). Each image illustrates how a specific predictor affects the accuracy and output of the model. The irradiation time is the most critical factor in extracting phenolic compounds from the plant material during MAE [62]. The extraction process typically comprises two stages and is exponential. While the mass transfer phenomenon in the second phase slowed, suggesting that the diffusion process was completed, the extraction yield in the first phase grew quickly [63]. It is also demonstrated that the change in the mass transfer mechanism accounts for two phases, showing that this exponential behavior is not exclusive to MAE. This demonstrates how the energy source regulates the rate at which each phase emerges and how the extraction process is linked to the solvent's and solute's physicochemical behavior [64]. The impact of microwave irradiation time on TPC, TFC, and DPPH was explored across a range of 4–8 min maximum with intervals. Maximum TPC, TFC, and antioxidant potential in terms of DPPH of *M. arvensis* occurred within 6 min. The optimum yield of bioactive compounds from plant material is enhanced by the optimal microwave power and biomass amount per unit volume of solvent during extraction. The selected microwave power range was 160–320 W, and the yield was inclined up to 240, after which the yield began to fall. The amount of biomass in a solvent accelerates the diffusion process of phyto-compounds. The yield of phytochemicals begins to decline up to a certain point when solute molecules get saturated in nearby solvents [44]. The biomass amount from 0.5-1.5 g was selected during the extraction. The significant yield of TPC, TFC, and DPPH was recorded as 124 mg GAE/g, 79 mg QE/g, and 90 % inhibition at 6.0 min, 240 W with 1.0 g biomass there was no appreciable increment in yield observed up to 8 min, 320 W and 1.5 g according to experimental data given in Table 1. The cumulative impact of all applied variables on predictors has been shown in Fig. 9 (b). This graph shows the mutual effect of two variables on predictors simultaneously. Notably, in 6 min combined at 240 W, the TPC (108 mg GAE/g), TFC (68 mg QE/g), and DPPH (80 % inhibition) reached their utmost values. Similar trends are observed at 6 min combined with 1.0 g biomass, 108 mg GAE/g, 70 mg QE/g, and 81 % inhibition achieved for TPC, TFC, and DPPH, respectively. Furthermore, the combination of biomass (1.0 g) and microwave power (240 W) shows the highest TPC (104 mg GAE/g), TFC (64 mg QE/g), and DPPH 84 % inhibition.

The 3D cumulative impact of the feature effects on the predictions is shown in Fig. S5. The three-dimensional (3D) cumulative impact model is utilized to visualize and analyze the effects of various feature variables on prediction outcomes. In this context, the features, such as time, microwave power, and biomass, are plotted along the three axes of the 3D space, allowing researchers to observe how changes in each feature influence the predicted values. This visualization provides insights into interactive effects, enabling the identification of complex relationships among the predictors. For instance, one can analyze how varying microwave power in conjunction with different biomass levels influences the overall predictions over time. By examining the cumulative impact represented in this 3D space, patterns emerge that may indicate optimal combinations of feature variables for enhancing extraction efficiency or other desired outcomes.

#### 3.3.3 Feature importance study

**Fig. 10:**
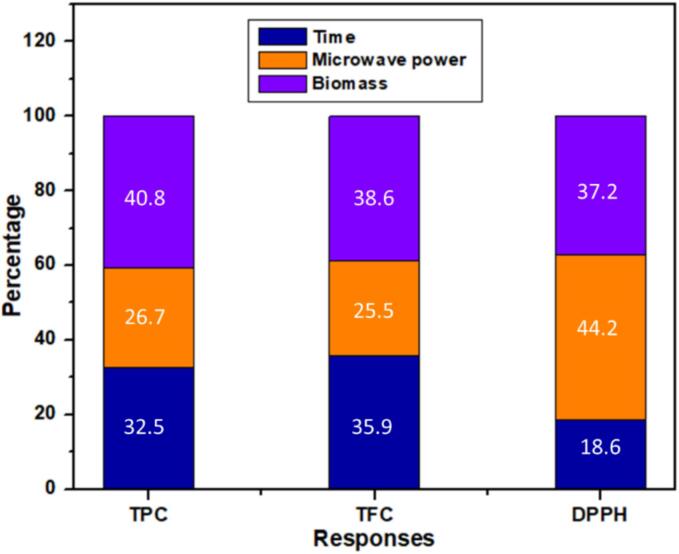
The average feature's importance with different machine learning algorithms.

**Fig. 11:**
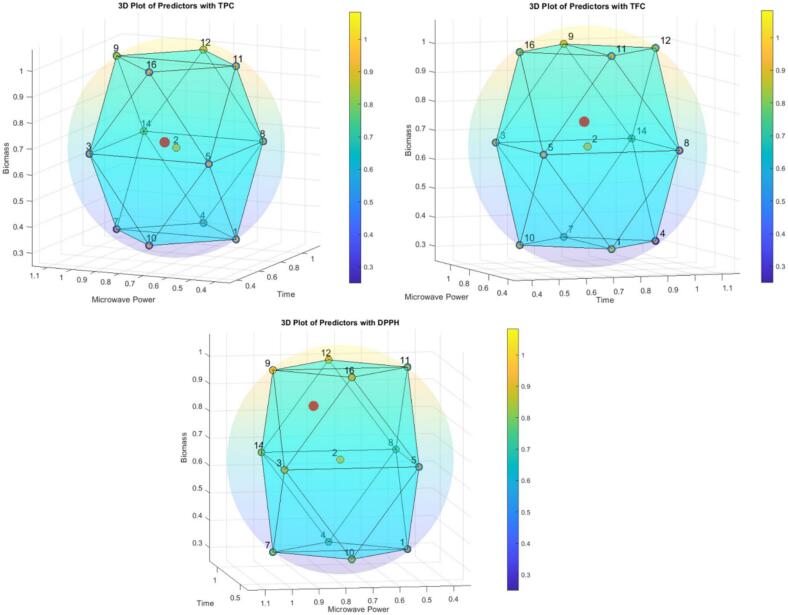
3D Radar plots showing the performance of predictors for different features.

The average feature’s importance is shown in Fig. 10, and in the case of TPC and TFC, the most significant feature is biomass quantity, which contributed 40.8 % and 38.6 %, respectively. In comparison, microwave power contributed more (44.2 %) for DPPH and less for TPC (26.7 %) and TFC (25.5 %). Time contributed 32.5 %, 35.9 %, and 18.6 % for TPC, TFC and DPPH, respectively. The permutation feature’s importance distribution [65] and the model features importance distribution methods used to investigate the effects of predictors, including time, microwave power, and biomass, on the response variable. The critical distribution of the permutation feature and model feature importance distribution are shown in the pie chart in Fig. S6. The CatBoost features importance distribution results displayed on the right side, and TPC and TFC contents (41.1 %) are highly influenced by biomass amount and then time (33.5 %). At the same time, microwave power is the least important factor (25.5 %) for TPC and TFC. The optimized amount of biomass in a unit volume of solvent is significant for effectively extracting bioactive components. [66]. However, for DPPH, the most crucial aspect is microwave power (46.3 %), followed by biomass (35.5 %), and the least critical aspect is time, which is 18.2 %. A distinct approach, permutation feature importance distribution, was employed to verify the accuracy of the feature significance rankings. The feature importance findings show slight variation in the three predictors versus responses, indicating that they are all about the same level of importance. Optimization of predictors with respect to responses is shown in Fig. 11. The central point indicated by red dot show the maximum yield of TPC, TFC and DPPH.

### 3.4 Antimicrobial testing

Table 5 shows the antimicrobial effects of the optimized extracts by well-diffusion method against selected bacterial and fungal strains. In antibacterial assay, larger inhibitory zones (*S. aureus* = 25.5 ± 1.4 mm and *E. coli* = 23.5 ± 1.4 mm) with MICs (*S. aureus* = 50 ± 2.5 µg/mL and *E. coli* = 100 ± 1.5 µg/mL) are observed with DES and aqueous methanolic extract showed 20.0 ± 0.4 mm inhibitory zone with MIC 100 ± 2.5 against *S. aureus* and 19 ± 0.8 mm inhibitory zone with 200 ± 5.0 µg/mL MIC value. Similarly, during antifungal evaluation, the eutectic extract exhibited superior inhibition zones (*F. solani* = 22.5 ± 1.4 mm and *A. niger* = 23.5 ± 0.8 mm) with MIC (*F. solani* = 100. ± 0.4 µg/mL and *A. niger* = 200 ± 0.5 µg/mL). The inhibition zones 14.5 ± 1.5 mm with 200 ± 1.5 µg/mL MIC against *F. solani* and 13.5 ± 1.5 mm with 100 ± 1.5 µg/mL against *A. niger* were observed with methanolic extracts. Overall, the findings confirmed that all plant extracts have antimicrobial action but not as much as Terbinafine and Rifampicin. Since DESs can dissolve a wide range of metabolites, including antimicrobial agents, they are more effective against microorganisms. One of the most common and well-known flavonoids is quercetin, which has been shown to efficiently stop the development of a variety of drug-resistant Gram-positive and Gram-negative bacteria, fungi, and viruses. This inhibitory action is attributed to its ability to disrupt bacterial cell walls and cause membrane damage [67]. A phytochemical study identified phenolic and terpenoid components in the *M. arvensis* extract, including rosmarinic acid, oleanolic acid, ursolic acid, luteolin, and isoorientin. The extract's antibacterial action may be attributed to its phenolic and terpenoid content [68].

**Table 5:** The potential of optimized extracts of *M. arvensis* with ChCl:EG DES against bacterial and fungal strains.

	***S. aureus***				***E. coli***			
	**Inhibition zone (mm)**		**MIC (µg/mL)**		**Inhibition zone (mm)**		**MIC (µg/mL)**	
**Extracts**	ChCl:EG	MeOH:H_2_O	ChCl:EG	MeOH:H_2_O	ChCl:EG	MeOH:H_2_O	ChCl:EG	MeOH:H_2_O
	25.5 ± 1.4	20.0 ± 0.4	50 ± 2.5	100 ± 2.5	23.5 ± 1.4	19 ± 0.8	100 ± 1.5	200 ± 5.0
**Rifampicin**	31 ± 2.4		25 ± 0.9		29 ± 2.4		12.5 ± 0.9	
	***F. solani***				***A. niger***			
	22.5 ± 1.4	14.5 ± 1.5	100 ± 0.4	200 ± 1.5	23.5 ± 0.8	13.5 ± 1.5	50 ± 0.5	100 ± 1.5
**Terbinafine**	25 ± 4.5		12.5 ± 0.9		28 ± 4.5		6.3 ± 0.9	

## 4 Conclusion

The potential of hydrogen-bonded supramolecular DES based on ChCl and EG as a sustainable alternative to traditional organic solvents for the phytochemical extraction process from *M. arvensis* was successfully demonstrated in this work. Compared to aqueous methanol (80 % methanol), the DES indicated a greater extraction yield of 314 mg/g at 240 W with 1.0 g biomass. Our study carried on a new level, including machine learning techniques, specifically the CatBoost model, which allowed for reliable phytochemical content prediction. Through coupling the capabilities of CatBoost, the research effectively managed complex, non-linear relationships within the data, resulting in more precise and instructive outcomes. The high R^2^ value (1.00) for TPC, TFC, and DPPH indicates an effective correlation between CatBoost’s predictions and the experimental data, which validates its accuracy and reliability for phytochemical analysis. The maximum absolute errors for TPC, TFC, and DPPH data were determined for the training and testing sets. In the training set, the maximum absolute errors were found to be 3.40 % for TPC, 2.799 % for TFC, and 2.40 % for DPPH. In the testing set, these errors were 2.599 % for TPC, 1.799 % for TFC, and 1.400 % for DPPH. Based on the permutation and model feature importance distribution, it was observed that biomass's contribution ranges from 35.5 % to 42.1 % towards generating the responses for TPC and TFC. Microwave power's contribution falls within the range of 24.5 % to 46.3 % for the response of DPPH. Additionally, the time's contribution ranges from 16.8 % to 33.5 % in generating the response for DPPH. This model's successful utilization highlights machine learning's transformative potential in advancing phytochemical research, providing a robust tool for enhancing future studies in this area. The DES extracts exhibited significant antibacterial activity against *S. aureus* (25.5 ± 1.4 mm) and *E. coli* (23.5 ± 1.4 mm) and prominent antifungal activity against *F. solani* (22.5 ± 1.4 mm) and *A. niger* (23.5 ± 0.8 mm) compared to aqueous methanol extracts. Our research suggests that sustainable DES-based microwave extraction, coupled with CatBoost, provides a viable way to extract phytochemicals from plant materials with more reliable data analysis. Based on our results, future research could examine the application of DES for the phytochemical extraction process from other plant species and look into the possible uses of these compounds in different sectors.

## Funding source

This research was supported by the Researchers Supporting Project number (RSPD2025R555), King Saud University, Riyadh, Saudi Arabia.

## Declaration of Generative AI and AI-assisted technologies in the writing process

During the preparation of this work the author(s) used ChatGPT in order to search some related machine learning data. After using this tool/service, the author(s) reviewed and edited the content as needed and take(s) full responsibility for the content of the publication.

## CRediT authorship contribution statement

**Zubera Naseem:** Writing – original draft, Methodology, Investigation, Conceptualization. **Muhammad Bilal Qadir:** Writing – review & editing, Visualization, Validation, Data curation. **Abdulaziz Bentalib:** Visualization, Funding acquisition. **Zubair Khaliq:** Writing – review & editing, Visualization, Supervision, Project administration. **Muhammad Zahid:** Supervision, Methodology, Investigation, Conceptualization. **Fayyaz Ahmad:** Validation, Supervision, Software, Resources. **Nimra Nadeem:** Visualization, Validation. **Anum Javaid:** Visualization, Validation.

## Declaration of competing interest

The authors declare that they have no known competing financial interests or personal relationships that could have appeared to influence the work reported in this paper.
