# Supplementary material for: Sustainable extraction of phytochemicals from Mentha arvensis using supramolecular eutectic solvent via microwave Irradiation: Unveiling insights with CatBoost-Driven feature analysis
Source: Ultrason Sonochem. 2025 Mar 4;115:107300. doi: 10.1016/j.ultsonch.2025.107300 (PMC11930105; doi:10.1016/j.ultsonch.2025.107300)
Supplement: Supplementary Data 1 [file mmc1.docx]

**Supplementary Information**

**Sustainable Extraction of Phytochemicals from Mentha arvensis Using Supramolecular Eutectic Solvent via Microwave Irradiation: Unveiling Insights with CatBoost-Driven Feature Analysis**

Zubera Naseem^1^, Muhammad Bilal Qadir^1^, Abdulaziz Bentalib^2^, Zubair Khaliq^1^, Muhammad Zahid^3^*, Fayyaz Ahmad^4^*, Nimra Nadeem^1^, Anum Javaid^5^,

^1^ Department of Textile Engineering, National Textile University, Faisalabad 37610, Pakistan

^2^ Department of Chemical Engineering, College of Engineering, King Saud University, P.O. Box 800, Riyadh 11421, Saudi Arabia

^3^ Department of Chemistry, University of Agriculture Faisalabad, 38040, Pakistan

^4^ Department of Applied Sciences, National Textile University, Faisalabad 37610, Pakistan

^5^ Shanghai Jiao Tong University Minhang Campus School of Materials Science and Engineering

**Evaluation of phytochemicals**

**Determination of TPC**

The TPC was determined by a modified Folin-Ciocalteau's (FC) reagent procedure. Three milliliters of saturated Na_2_CO_3_, one milliliter of the extract, and five milliliters (10 percent) of FC reagent were combined and put into incubation for 2 h at RT. A calibration curve of gallic acid at values ranging from 200–1000 µg/mL in ethanol was created using a similar methodology. The appropriate solvent was utilized as the blank for analyzing the absorbance at 730 nm using a CECiL-7200 spectrophotometer.

**Determination of TFC**

The TFC was determined by a significant modification to the aluminum chloride colorimetric technique. Analytical grade quercetin was diluted in methanol to produce a calibration curve that could be used as a reference for measuring flavonoid content. The range of 0-100 µg/mL was chosen. In the analysis, equal parts of potassium acetate (0.1 M) and AlCl_3_·6H_2_O (2%) solution were combined with a diluted extract of quercetin (2.0 mL). The mixture was incubated for 30 min at RT before measurement of absorbance at 415 nm with a CECiL-7200 spectrophotometer.

**Evaluation of antioxidant potential (DPPH)**

The extract’s potential to scavenge the free radicals was determined with the DPPH radical assay method, with a few minor modifications. Specifically, 1.0 mL of the extracts (ranging from 0-1.0 mg/mL) was with 2 mL of a DPPH solution in ethanol (0.2 mM) and incubated for 30 min at 37°C. The measurement of absorbance was done after incubation (517 nm) using a CECiL-7200 spectrophotometer. The % inhibition was utilized to calculate the free radical scavenging potential.

**
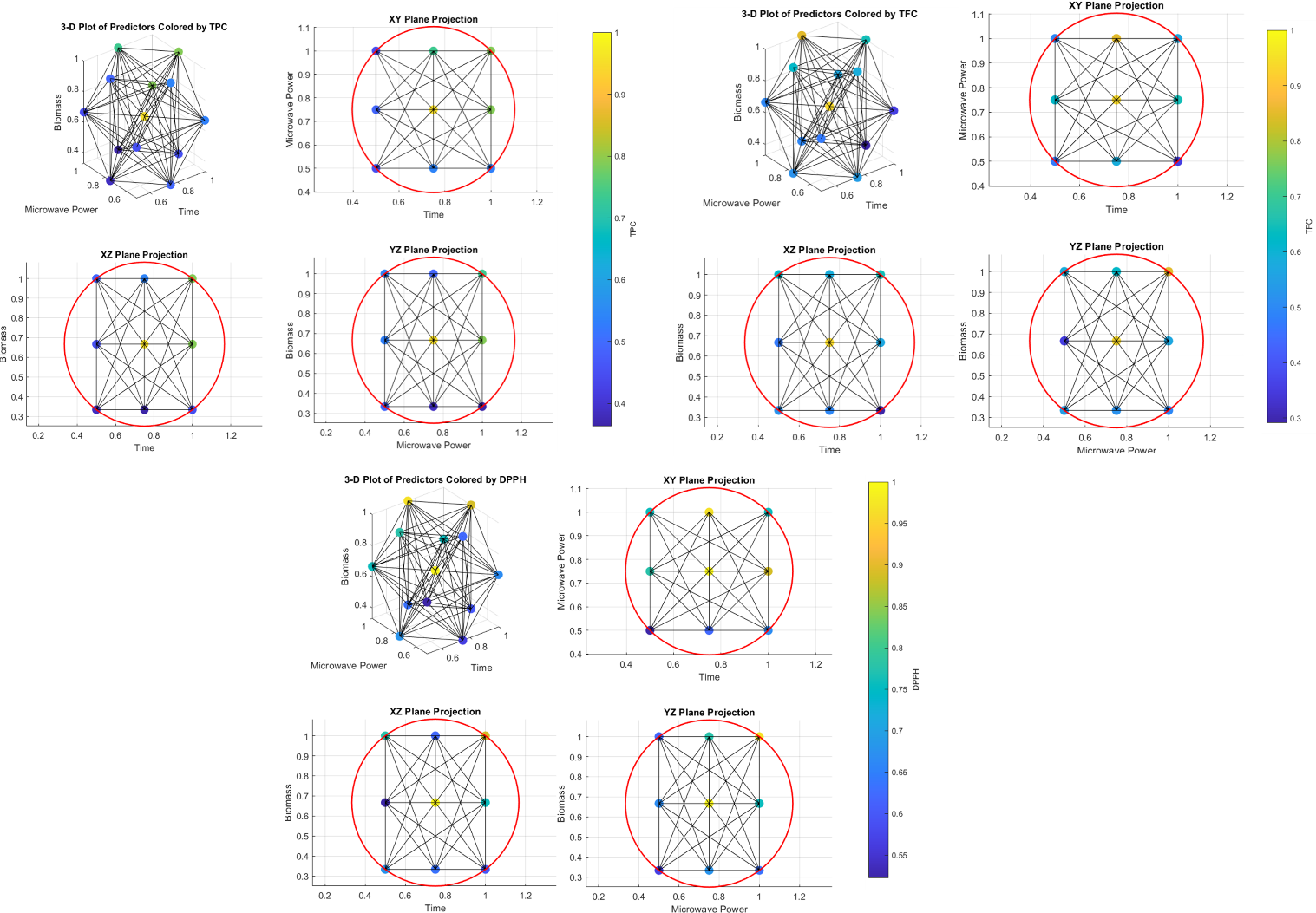
**

**Figure S1: The 3D geometrical representation of predictors in x, y, and z planes**


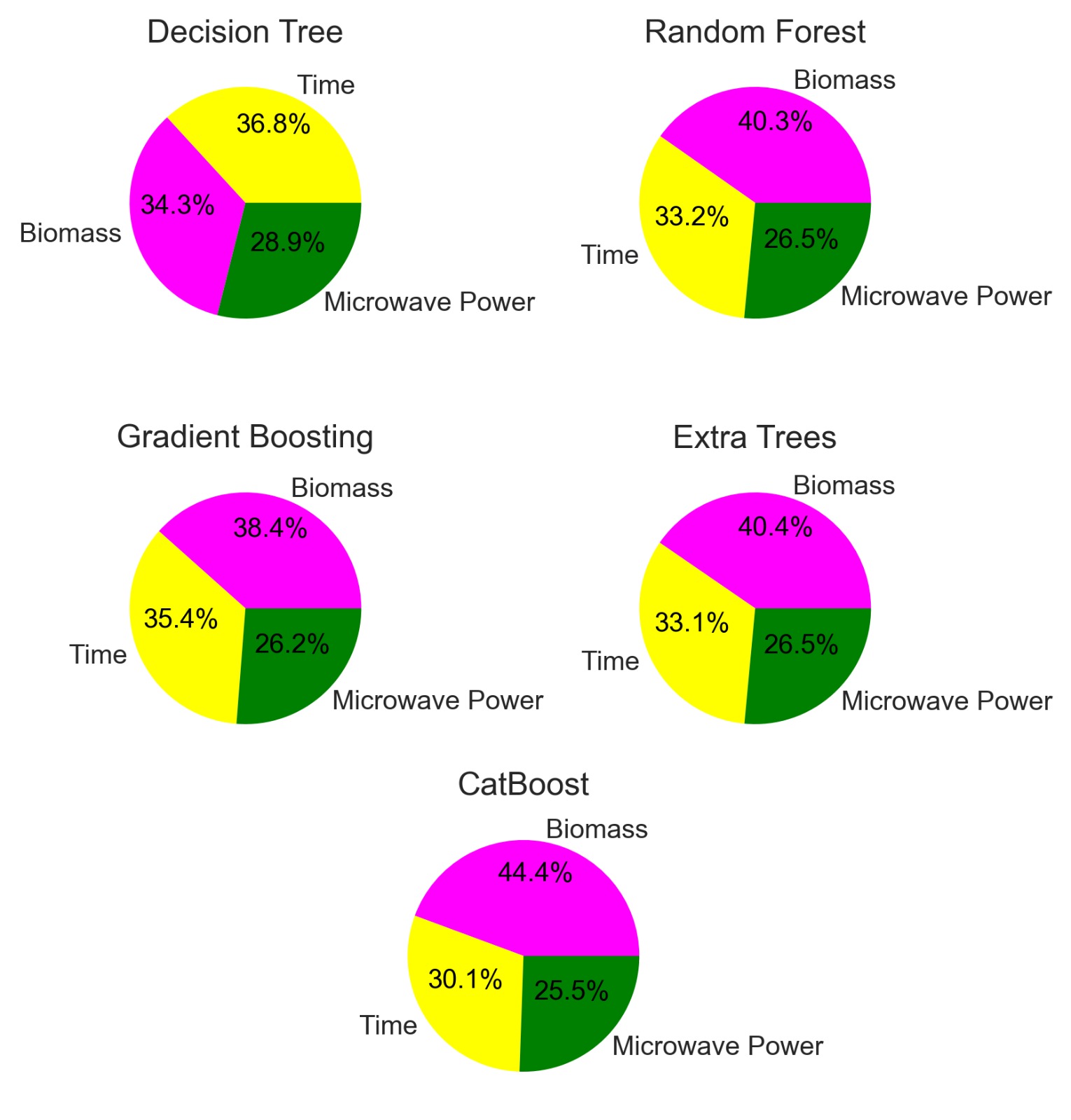


**Figure S2: Feature importance of CatBoost model over Decision Tree, Random Forest, Gradient Boosting, and Extra Trees for TPC**


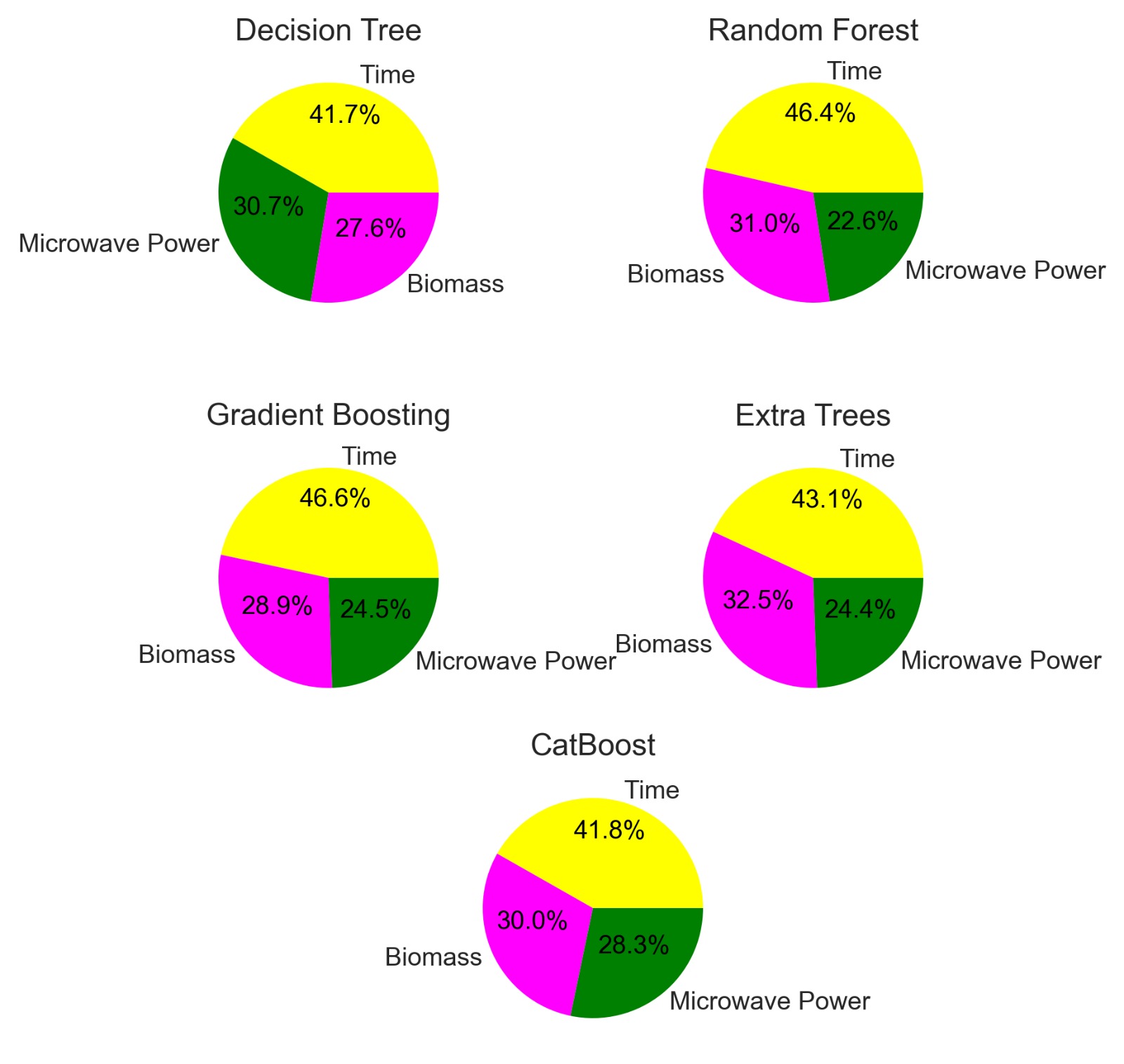


**Figure S3: Feature importance of CatBoost model over Decision Tree, Random Forest, Gradient Boosting, and Extra Trees for TFC**


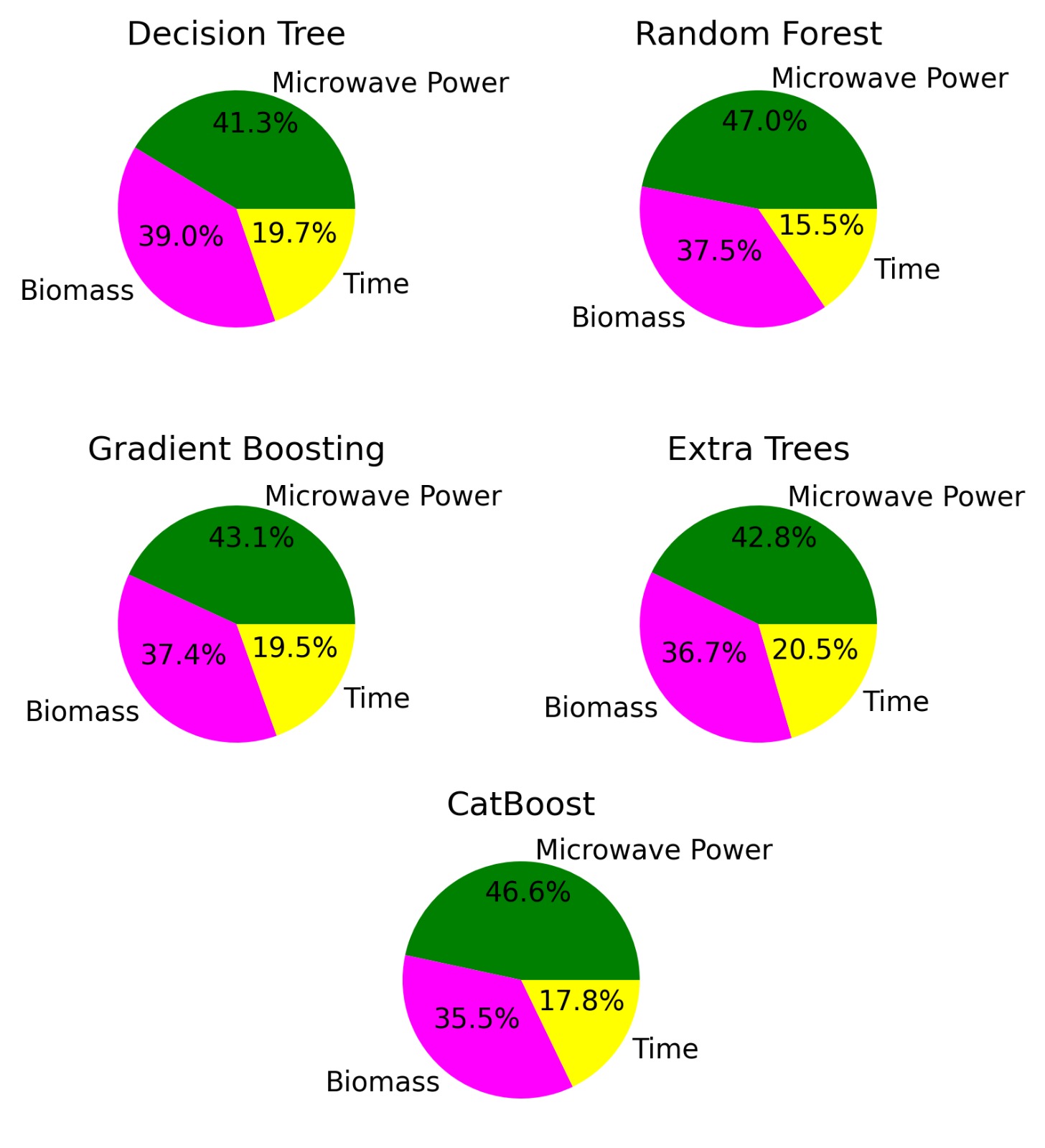


**Figure S4: Feature importance of CatBoost model over Decision Tree, Random Forest, Gradient Boosting, and Extra Trees for DPPH**

**Antimicrobial activities**

**Antibacterial activity**

In a nutrient broth medium, two bacterial strains were grown and incubated for 24 h at 37°C with constant agitation at 120 rpm. After cultivation, the cultures were maintained at a temperature of 2 to 8°C. Petri plates were laminar-aired until they reached room temperature following autoclaving and cleaning. After being suspended in 1000 mL of distilled water, the 28 g of nutritious agar was autoclaved for 30 min (121°C and 15 pounds of pressure). After the medium reached a temperature of 40 to 50°C, 1000 µL of the bacterial culture was added and thoroughly mixed. Subsequently, approximately 50 mL of the mixture was transferred into each petri dish and solidified. Using a sterile cork borer (4 mm), wells were created in the solid medium, and 100 µL (100 mg/mL) of the extracts were pipetted into each well using a micropipette. Each petri plate was filled halfway with 100 mg/mL of rifampicin as standard. After that, the plates were incubated for a full day at 37°C. Using a zone reader, the diameter of inhibitory zones surrounding the well was measured in millimeters to determine the antibacterial activity.

***Minimum inhibitory concentration***

On the first row of a microtiter plate, 100 µL (800 µg/mL) of plant extract was added, and 50 µL of nutritional broth medium was added to the other wells. Decreased concentrations were achieved by serial two-fold dilutions, wherein 50 μL of *M. arvensis* extract solution in nutritional broth medium was added to each consecutive well. After that, 10 μL of the inoculum was introduced to each well, and the positive and negative controls were Rifampicin and water, respectively.. Following incubation (24 h at 37°C ), each well received a 10 μL resazurin solution indicator, which changes color from blue to pink to show the presence of bacteria. With the microtiter plate in place, an ELISA reader was used for absorbance measurement at 620 nm. The lowest concentration at which bacterial growth was not visible was identified as the minimum inhibitory concentration (MIC) value.

**Antifungal activity**

The fungal strains were cultured for 72 hours at 28°C in dextrose broth, or until the fungus started to multiply and the soup turned murky. Following that, the fungal inoculum was stored in a cold cabinet between 2 and 8°C for further analysis.
To prepare the growth medium, 1000 milliliters of distilled water were mixed with 39 grams of potato dextrose agar. The mixture was then placed under 15 pounds of pressure and autoclaved for 30 min at 121°C. The medium was chilled to 45–50 °C using a laminar airflow cabinet, and then 1000 µL of the fungal culture was added and properly mixed. After that, 50 mL of the medium was added to sterile petri dishes and let to harden. After making wells in the solid medium using a sterile cork borer (4 mm), 100 µL of *M. arvensis* extract was carefully pipetted into each well. Terbinafine served as the positive control in the assessment of antifungal activity during a two-day incubation period at 28 °C. The millimeter diameters of the inhibitory zones demonstrated the antifungal effectiveness of the *M. arvensis* extracts. To minimize errors and ensure data accuracy, the antifungal tests were repeated three times, preserving accuracy and reliability in the experimental results.

***Minimum inhibitory concentration***

A microtiter plate was initially row-filled with 100 µL (800 µg/mL) of *M. arvensis* extract, then the remaining wells were filled with 50 µL of nutritious broth medium. The plant extract solution in nutrient broth medium was gradually diluted two more times until 50 μL of the solution was present in each well. Next, 10 μL of fungal inoculum was introduced into every well. Terbinafine was used as the positive control and water as the negative control. An ELISA reader was used to assess the absorbance at 520 nm after a 48 h incubation period at 28°C. The minimum concentration of fungal growth inhibited by the extract was determined to be its Minimum Inhibitory Concentration (MIC) value.

**
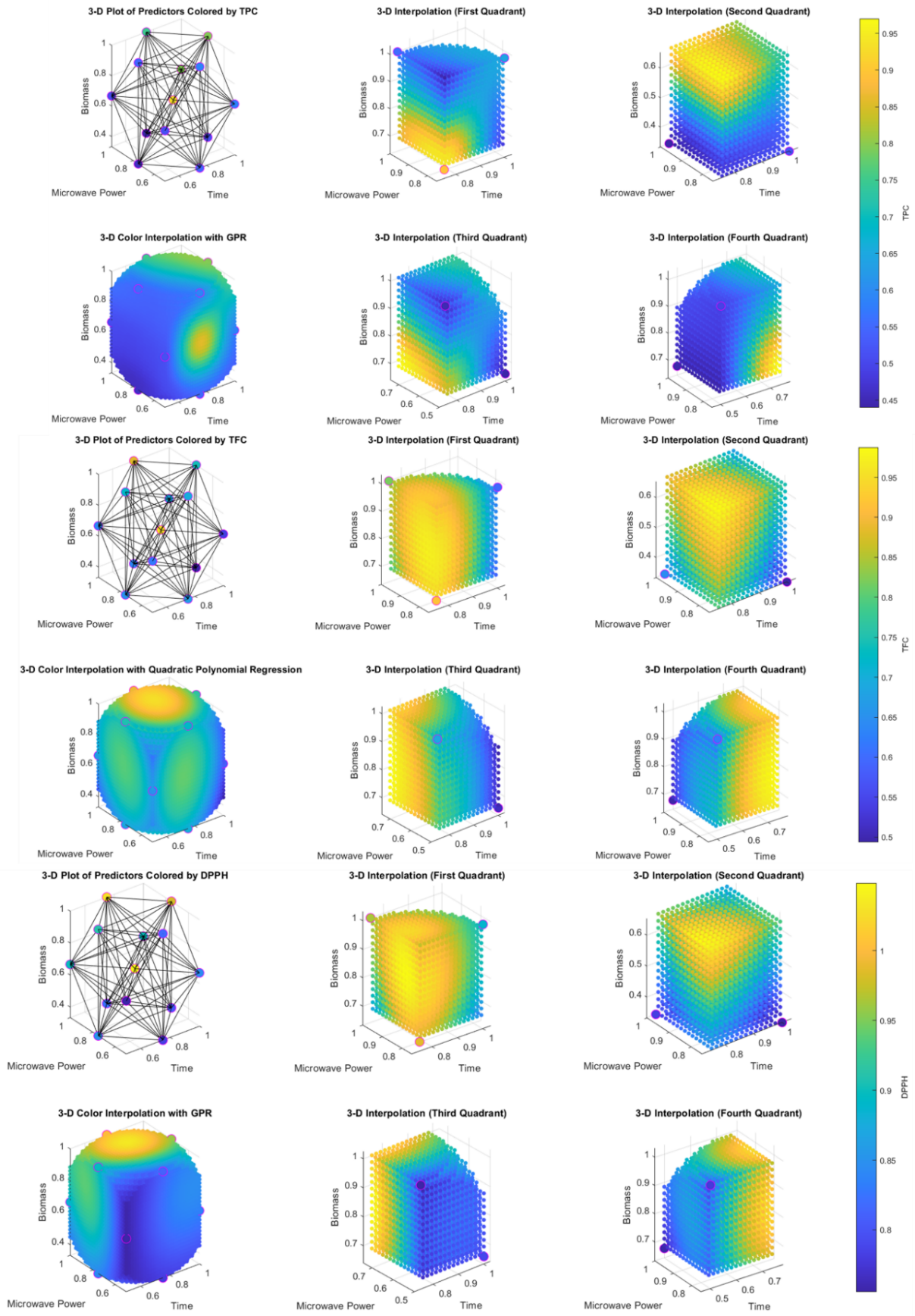
**

**Figure S5: The 3D cumulative impact of the feature effects on the predictions for TPC, TFC and DPPH of M. arvensis with ChCl:EG DES**

**
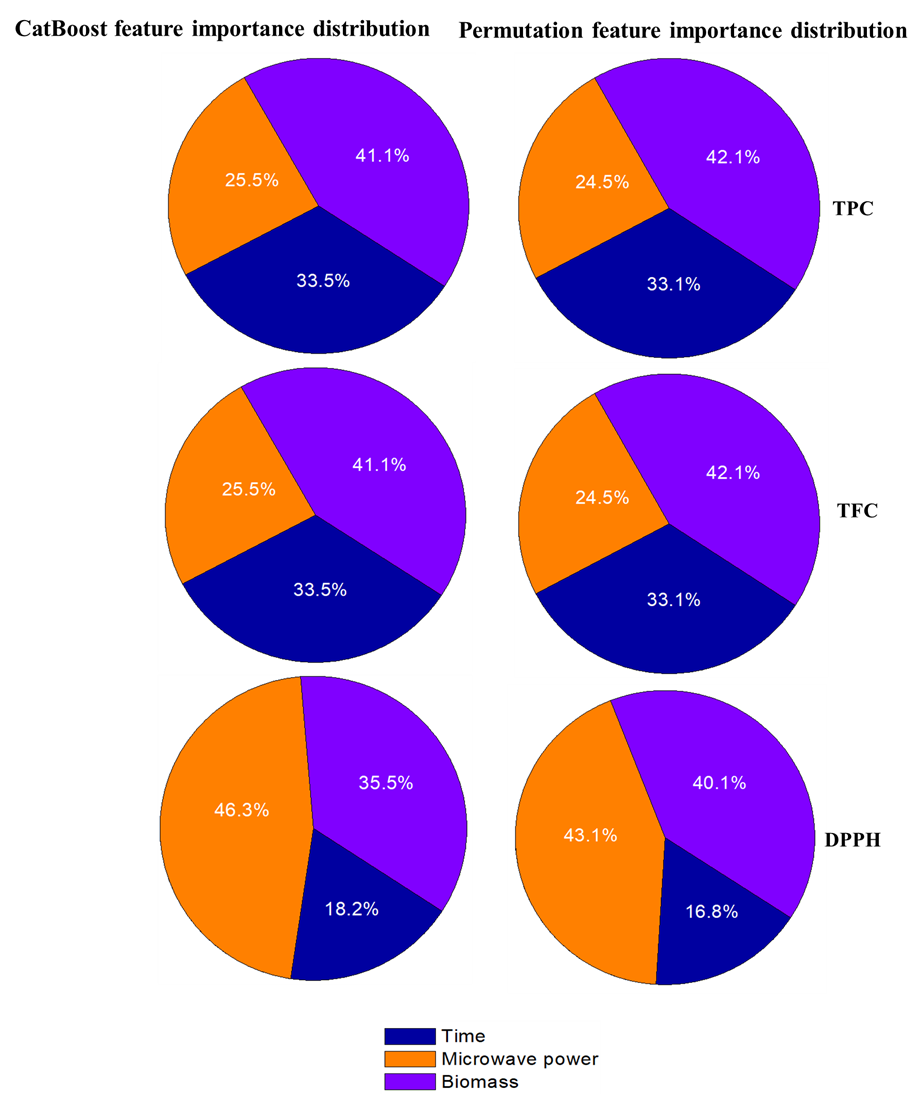
**

**Figure S6: The importance of time, microwave power, and biomass is evaluated by the CatBoost feature importance and permutation feature importance for TPC, TFC and DPPH of M. arvensis with ChCl:EG DES**
